# Reply: Changes in BMI after treatment of testicular cancer are due to age and hormonal function and not chemotherapy

**DOI:** 10.1038/sj.bjc.6601179

**Published:** 2003-09-09

**Authors:** C Nord, S D Fosså

**Affiliations:** 1 Department of Clinical Research, The Norwegian Radiumhospital, Montebello N-0310 Oslo, Norway

**Sir**,

We would like to clarify the results in ‘Excessive annual BMI increase after chemotherapy among young survivors of testicular cancer’ as a reply to Huddart *et al*'s Letter to the Editor.

In some agreement to Huddart *et al*'s results, our study showed no influence of chemotherapy or any other treatment type on BMI compared to our healthy control material (Controls). Our results from the multivariate regression analysis indicated that in survivors of testicular cancer (TCSs), chemotherapy had an impact on the annual BMI increase independent of age and that the surgery group displayed no significant annual BMI increase (Nord *et al*, 2003). However, it only explained 8% of our observations as compared to the normal population, thus indicating a multifactorial background for the annual BMI increase. In Huddart *et al*'s publication, the comparison to an age-matched cohort from the normal male population is lacking (Huddart *et al*, 2003). This is important as we, indeed, did not find significant BMI increase comparing the surgery group with the chemotherapy group. We admit that it is a weakness in our study that so few other explanatory factors were included in the regression analysis such as gonadal hormones, smoking, exercise and eating habits, which all have well-documented influences on BMI.

We still conclude that young TCSs, especially after chemotherapy, are at risk of excessive annual BMI increase as compared to the normal population (*P*=0.013), but our results do not show any impact on BMI, measured once in a cross-sectional study.
